# Investigation on the Pathological Mechanism of Frequent Exacerbators With Chronic Obstructive Pulmonary Disease Based on the Characteristics of Respiratory Flora

**DOI:** 10.3389/fmed.2021.816802

**Published:** 2022-01-20

**Authors:** Li Ke, Luo Chen, Yuan Yaling, Gao Can, Lin Jun, Zhang Chuan

**Affiliations:** ^1^Department of Laboratory Medicine, Chongqing the Seventh People's Hospital, Chongqing, China; ^2^Department of Respiratory and Critical Care, Chongqing the Seventh People's Hospital, Chongqing, China

**Keywords:** COPD frequent exacerbators, COPD infrequent exacerbators, bacterial flora analysis, Veillonella parvula, COPD

## Abstract

Chronic obstructive pulmonary disease (COPD) is a common obstructive respiratory disease characterized by persistent respiratory symptoms and limited airflow due to airway obstruction. The present study investigates the distribution characteristics of respiratory tract flora in both frequent and infrequent exacerbators of COPD. The 16S sequencing technique was adopted to differentiate the inherent differences of respiratory tract flora between frequent exacerbators and infrequent exacerbators. Additionally, cell counting kit 8 (CCK8), lactate dehydrogenase (LDH) test, flow cytometry, enzyme-linked immunosorbent assay (ELISA), and western blot were carried out in human bronchial epithelial cells cultured *in vitro* and the regulatory effects of differential flora were verified. The results revealed that the observed species index, Chao1 index, and the ACE estimator of COPD frequent exacerbators were markedly higher than those of COPD infrequent exacerbators. The top five strains of COPD frequent exacerbators included g_Streptococcus (15.565%), g_Prevotella (10.683%), g_Veillonella (6.980%), g_Haemophilus (5.601%), and g_Neisseria (4.631%). Veillonella parvula generated obvious cytotoxicity and substantially reduced the activity of human bronchial epithelial cells (*p* < 0.01). Furthermore, the results of flow cytometry indicated that the proportion of human bronchial epithelial cells in both the S phase and G2 phase decreased following Veillonella parvula treatment indicated that Veillonella parvula inhibited cell proliferation. Meanwhile, being treated using Veillonella parvula, the expressions of interleukin-1 (IL-1), IL-6, Tumor Necrosis Factor α (TNF-α), and p-nuclear factor kappa B (NF-κB) of the cells were increased markedly (*p* < 0.01). Taken together, the current research demonstrated that the relative abundance of Veillonella in COPD frequent exacerbators was higher than that of infrequent exacerbators. Veillonella parvula activated the inflammatory pathway, ultimately destroyed the cell viability, and greatly impaired the activity of human bronchial epithelial cells, thereby inhibiting cell proliferation.

## Introduction

Chronic obstructive pulmonary disease (COPD) has emerged as the third most common cause of death globally, and it is estimated to become the fifth financially burdened disease (1, 2). The acute exacerbation of COPD is mainly responsible for this burden. Clinically, patients with COPD are divided into frequent exacerbators and infrequent exacerbators. The former is defined as those who suffer from twice of exacerbated episodes within 12 months, and the morbidity and mortality are higher than infrequent exacerbators (3, 4). Increasing evidence has indicated that COPD frequent exacerbation is intimately associated with the unbalanced normal bacterial flora colonization in the respiratory tract, but there are obvious regional differences in the distribution characteristics of the bacterial flora, and the relationship between the airway flora and the phenotype of exacerbation is unclear (5–7).

The next-generation sequencing technique is available for simultaneous sample detection of thousands of bacteria covered in the database (5–7). The 16S sequencing refers to selecting one or several variant regions of 16S rDNA, selecting universal primers for PCR amplification of environmental sample microorganisms, then performing high-throughput sequencing on the PCR products, and comparing the obtained sequencing data with the existing 16S rDNA database. The core is species analysis, such as the types of microorganisms, the relative abundance of different species, the species differences between different groups, and system evolution. Researchers have introduced this approach to detect respiratory disease specimens obtained from the respiratory tract, hoping to discover the correlation between the distribution of bacterial flora and the occurrence and development of diseases (8, 9). These studies have covered disorders of bronchial asthma, cystic fibrosis, and COPD, which contributes a lot in broadening our recognition and understanding of respiratory etiology. Results of bacterial flora analysis have revealed that Streptococcus, Prevotella, and Haemophilus influenzae are common bacteria in the respiratory tract (10). Fusobacterium and Pseudomonas are also colonized in the lower respiratory tract (11, 12). Based on existing studies, during the period of COPD exacerbation, the α-diversity of the flora decreases compared with the stable period, and the ratio of Proteobacteria/Firmicutes increases. Moreover, during the period of acute exacerbation, the relative abundance of Moraxella is significantly increased, whereas Haemophilus presents no significant increase compared with the stable period (13–15). Investigation on respiratory microbiome began in 2010 and remains in an early stage. Moreover, related data are insufficient globally, most research has limited samples, and the research conducted in China is even rarer. Therefore, many biomarkers are still under the stage of identification and more further cohort data from different regions are to be validated (16).

This project explores the distribution characteristics of the respiratory tract flora in both COPD frequent exacerbators and infrequent exacerbators in the local region and to identify the inherent differences in the respiratory flora between COPD frequent exacerbators and infrequent exacerbators. We attempted to determine the risk-related target flora with COPD frequent exacerbators and verified in human bronchial epithelial cells cultured *in vitro*. The present project was expected to open up a novel approach for the prevention and treatment of COPD frequent exacerbators.

## Materials and Methods

### Clinical Data

This research was a prospective and observational study, which was approved and documented by the Medical Ethics Committee of Chongqing the Seventh People's Hospital (approval number: 202019). COPD frequent exacerbators (48 cases) and COPD infrequent exacerbators (32 cases) were recruited from Chongqing Seventh People's Hospital, and sputum samples were collected.

### Collection of Sputum Specimens

The collection procedures were strictly performed following the aseptic operation requirements. The surface of fresh sputum was removed using a sterile cotton swab to avoid being mixed with other sundries. The inner layer of the sputum sample was obtained and transferred into a sterile cryotube using a new cotton swab. Immediately after sample collection, the specimens were stored in a refrigerator at −20°C, transferred to the laboratory within 24 h, and kept in a refrigerator at −80°C for later use.

### DNA Extraction and Purification of Bacterial Flora in Sputum Samples

A QlAamp DNA Stool Mini Kit (Qiagen Company, Germany) was employed to extract DNA, and a Qubit Fluorometer Kit (Life Technologies, Carlsbad, CA, USA) was adopted to detect DNA concentration, and 1% agarose gel electrophoresis was performed to detect sample integrity. Taq enzyme (Sangon Biotech, Shanghai, China) was used to amplify selected regions (V3–V4 regions), PCR primers were 338F (5'-ACTCCTACGGGAGGCAGCA-3') and 806B (5'-GGACTACHVGGGTWTCTAAT-3'). After passing the 1% agarose gel electrophoresis test, the samples were delivered for high-throughput sequencing of 16S analysis of bacteria in the laboratory.

### High-Throughput Sequencing of 16S Analysis of Bacteria

Illumina Hiseq 2500 (Illumina, San Diego, CA, USA) sequencing platform was employed for PE250 library construction and sequencing, using pair-end sequencing (Pair-end) method, each sequence generates 250 reads from the 5′ and 3′ end. The raw data obtained for sequencing were trimmed and filtered before being applied for analysis, and the low-quality reads were filtered out and only valid data were retained. The reads were spliced into Tags using overlapped valid data, and the target fragments were obtained by further filtering. Using 97% similarity, the Tags were clustered into operational taxa (OTU) and compared with the 16S database of known species for OTU species annotation, and the community composition of each sample was subsequently obtained.

### Bacterial Flora Analysis Method

QIIME2 software (Version 2.0.2, https://qiime2.org/) was used to analyze the microbiome sequence data. The 16S rRNA representative sequence was constructed using the silva-138-99 (QIIME 2 2020.8 Species Annotation Database) provided by SILVA (https://www.arb-silva.de/) amplicon database with a similarity threshold of 97%. To remove the potential signals in the contaminants, we removed the analytical taxa with a relative abundance of 10% in the sample and the OTUs with a total abundance of 1 in the sample from the analytical taxa. The remaining reads were aggregated into 3,942 OTUs.

Alpha diversity used Observed species index (the actual number of OTUs observed), Chao1 index (estimated total number of OTUs contained in the sample), ACE index (estimated total number of OTUs contained in the sample), Shannon index (estimated microbial community diversity), and The Simpson index (estimated microbial community diversity) to compare the Alpha-diversity of the microbial community in the HIV group and the Norma group. Pie charts and histograms were used to show the proportion of genus-level microorganisms in the sample, that is, the relative abundance of microorganisms. Linear discriminant analysis effect size (LEfSe) was a method of discovering biomarkers to determine the best characteristic genus for each study group. The LEfSe score measures and analyzes the consistency of the relative abundance differences of taxa in each group. The higher the score, the higher the consistency. We considered that the groups with Linear Discriminant Analysis (LDA) score >4 and *p* < 0.05 were significant. The different species at the genus level and the provided physiochemical factors were applied to calculate the Spearman correlation between the species and the physiochemical factors. R software (4.1.0, https://www.r-project.org/) psych package/The reshape2 package/ggplot2 package was used to plot heat maps to reveal the essential connection between both aspects.

### Culture of Human Bronchial Epithelial Cells

Human bronchial epithelial cells were purchased from American Type Culture Collection and cultured in a complete medium containing 10% fetal bovine serum (FBS) and 1% penicillin-streptomycin. The cells were categorized into a control group and a model group. Following 24 h of inoculation, the cells in the model group were added 50 μl of active Veillonella parvula (ATCC #10790) and 50 μl of bacteria through high-temperature sterilization to the control group. After 24 h, the effect of Veillonella parvula on human bronchial epithelial cells was determined.

### Cell Counting Kit (CCK8) Assay

The cells were placed in a 96-well plate with 100 μl per well, and there were three multiple wells in each group. Cell modeling and grouping were subsequently managed as per the abovementioned conditions, and the cells were placed in a carbon dioxide incubator. After 24 h of incubation, the 96-well plate was taken out and 10 μl of CCK8 solution (AC11L054, Life-iLab, China) was supplemented to each well. The culture plate was kept in the incubator for 4 h and then terminate the culture. The absorbance of each well was determined at 450 nm using an enzyme-linked immunometric meter.

### Lactate Dehydrogenase (LDH Detection)

After the stimulation, the cell culture plate was centrifuged at 400 g for 5 min in a multi-well plate centrifuge. The supernatant was aspirated as much as possible. Subsequently, 150 μl of the LDH release reagent was added, which was supplied with a 10-fold PBS diluted kit, mixed well by moderately vibrating the culture plate, and then continued to incubate in the cell culture incubator for 1 h. Subsequently, the cell culture plate was centrifuged at 400 g for 5 min using the multi-well plate centrifuge. In total, 120 μl of the supernatant was obtained from each well, added to the corresponding well of a new 96-well plate, and proceeded to sample determination instantly. Of 60 μl LDH detection working solution (C0016, Beyotime, China) was supplied to each well, mixed evenly, incubated at 25°C in the dark for 30 min, and measured the absorbance at 490 nm. Cytotoxicity or mortality (%) = (absorbance of treated samples – absorbance of samples in control hole)/(absorbance of cells with maximum enzyme activity – absorbance of samples in control hole) × 100.

### Detection of Cell Cycles by Flow Cytometry

The cells were cultured in a 6-well plate. When the cell growth was reached 60–70%, they were aspirated into a 1.5 ml centrifuge tube after cell treatment and centrifuged at 2,000 rpm for 5 min. The cells were rinsed twice with PBS and centrifuged at 2,000 rpm for 5 min. Then, the cells were added in 1 ml of pre-cooled 70% ethanol in an ice bath, blown gently to mix well, and fixed at 4°C for 2 h. The cells were precipitated after centrifugation at about 1,000 rpm for 5 min, added about 1 ml of ice-cooled PBS for cell resuspension, centrifuged again, and carefully aspirated the supernatant. Of 0.5 ml propidium iodide staining solution was added to each tube with sample cells. Cell precipitation was suspended slowly and fully, warm bathed at 37°C for 30 min, and kept away from light. After that, the cells were stored either at 4°C or kept in an ice bath away from light. It is advisable to perform flow cytometry within 24 h after staining. Red fluorescence and light scattering were subjected to flow cytometry at an excitation wavelength of 488 nm.

### Enzyme-Linked Immunosorbent Assay (ELISA)

The interleukin-1 (IL-1β), IL-6, and Tumor Necrosis Factor α (TNF-α) ELISA kits (RX106152H, RX106126H, RX104793H, Ruixin BIO, China) were purchased from Ruixin Biotech, and the tests were carried out as per product instructions of use. The required plates were prepared firstly, marked standard wells and sample wells, added 50 μl of standard products of different concentrations to each standard wells and 50 μl of the sample to be tested to the sample wells. Detection antibody labeled by horseradish catalase 100 μl was supplemented to each standard solution well and sample well, respectively. Following the plate was blocked with sealing membrane, it was incubated at 37°C for 60 min. After the incubation was completed, discarded the liquid of the wells, dried it on a piece of absorbent paper, filled each well with washing solution, let stand for 1 min, discarded the washing solution, dried it again on a piece of absorbent paper, and repeated five times. Following the addition of substrates A and B 50 μl, respectively, to each well, incubation was performed at 37°C for 15 min in dark. When the incubation was completed, a termination solution at a quantity of 50 μl was added to each well. optical density (OD) value of each well was measured at 450 nm wavelength within 15 min.

### Western Blot (WB) Detects the Expressions of p-Nuclear Factor Kappa B (NF-κB) and NF-κB

Prepared gel was taken out from a refrigerator at 4°C and placed in an electrophoresis tank. Of 500 μg, total proteins were taken from each sample and mixed with 5 × Sodium dodecyl sulfate (SDS) loading sample buffer at a ratio of 4:1. The concentration of protein in the mixture was about 3.3 μg/μl, and the proteins were denatured by metal bath heating at 100°C for 6 min. The denatured total proteins of 60 g were selected for sample loading. After running at 80 V using spacer gel, the voltage was switched to 120 V and waited until the bromophenol orchid just covered the bottom of the gel plate without spilling out. The clip was loosed to level at the black side horizontally and placed sponge pad, filtering paper, gel, polyvinylidene fluoride (PVDF) membrane, filtering paper, and sponge pad in turn. The current was set at a constant 250 mA for 30 min. The membrane was taken out, marked well on both sides, washed in tris buffered saline with tween (TBST) for 1 min, and sealed with 5% skimmed milk blocking buffer at room temperature for 1 h. After sealing, the membrane was washed with TBST three times, 5 min each time. Primary antibody was diluted with primary antibody diluent at 1:1,000, incubated overnight at 4°C, and rinsed with TBST for three times, 10 min each. Secondary antibody was diluted using the blocking buffer, incubated for 1.0 h at room temperature, and rinsed with TBST for three times, 10 min each time. The ECL exposure solution was mixed with liquid A and B at a 1:1 rate and then evenly covered on the entire membrane. After reaction for 1 min, it was loaded in the exposure meter for detection. Antibodies used were as follows: p-NF-κB (3033T, CST, Danvers, MA, USA); NF-κB (10745-1-AP, Proteintech, Rosemont, IL, USA); GAPDH (A19056, Abclonal, China); HRP-Goat-anti-Rabbit (AS014, Abclonal, China).

### Statistical Analysis

SPSS 22.0 statistical analysis software was employed for data analysis, and the measurement index was expressed as mean ± SEM. A *t*-test was adopted for the data in conformity to normal distribution. The comparison of categorical count indexes was tested by the χ^2^ tests or Fisher's exact probability method. The hypothesis testing used two-sided testing to obtain test statistics and their corresponding *p* values, and *p* < 0.05 was considered as the standard of significant difference.

## Results

### Analysis of Microbial Diversity in Sputum Samples of COPD Frequent Exacerbators and Infrequent Exacerbators

**Figure 1:**
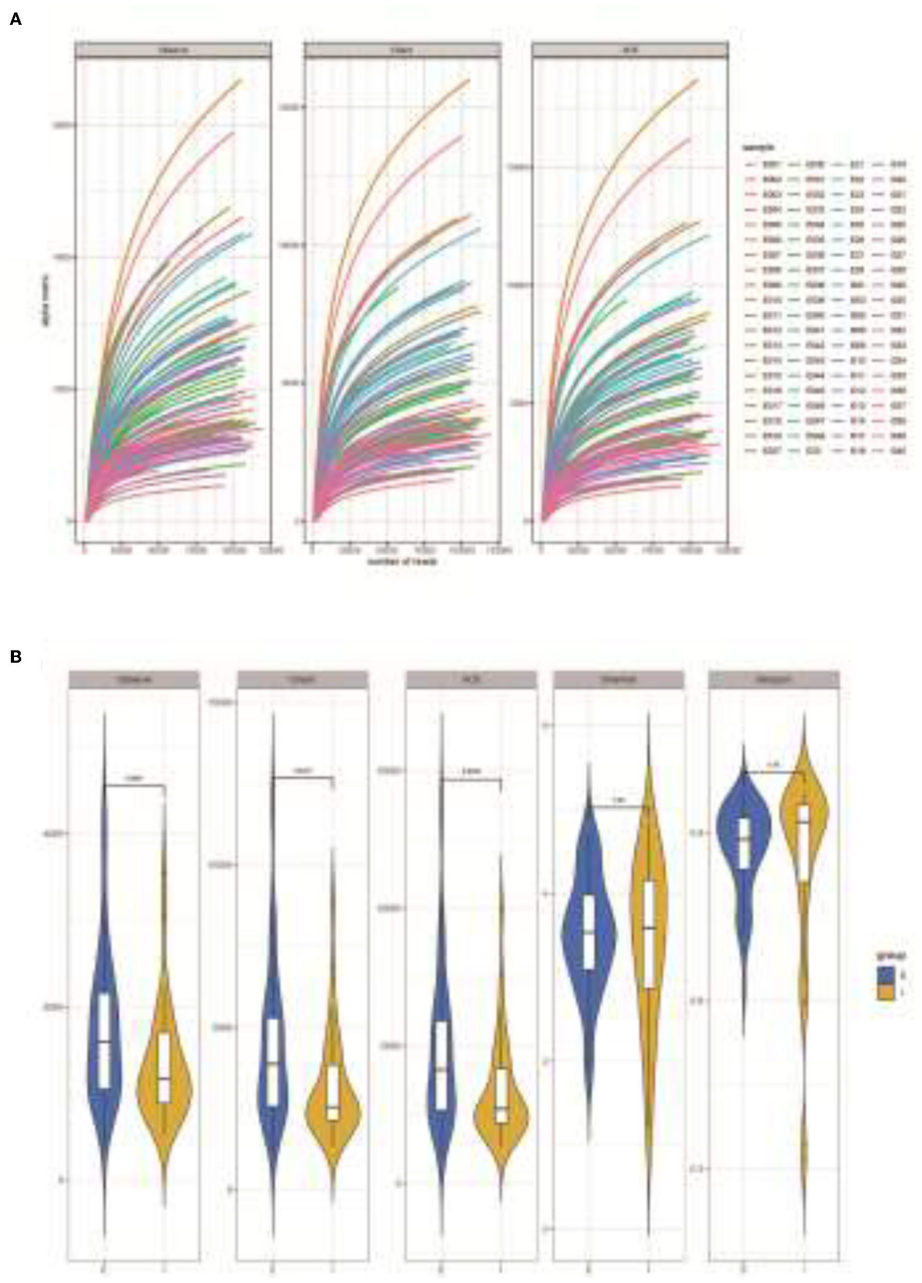
Analysis of microbial diversity in sputum samples of COPD frequent exacerbators and infrequent exacerbators. **(A)**, 16S sequencing dilution curve results. **(B)**, QIIME software was used to calculate the Alpha diversity index of both sample groups. E represented COPD frequent exacerbators; I represented COPD infrequent exacerbators. COPD, chronic obstructive pulmonary disease.

This study analyzed the sputum samples obtained from 48 COPD frequent exacerbators and 32 cases of infrequent exacerbators using the 16S sequencing technique. A total of 8,741,237 reads were collected from all samples, and 4,440 OTUs were obtained by clustering. Based on the dilution curve, the curve line had a tendency of going stable and the quantity of sequencing data was sufficient. It was therefore that the sequencing results of this project could reflect most of the microbial diversity information in the samples (Figure 1A). QIIME software was applied to calculate the index values of Alpha diversity in both groups of samples, and it was found that the Observed species index, Chao1 index, and ACE index of COPD frequent exacerbators were markedly higher than COPD infrequent exacerbators (*p* < 0.05; Figure 1B).

### Beta Analysis of Microbial Diversity in Samples

**Figure 2:**
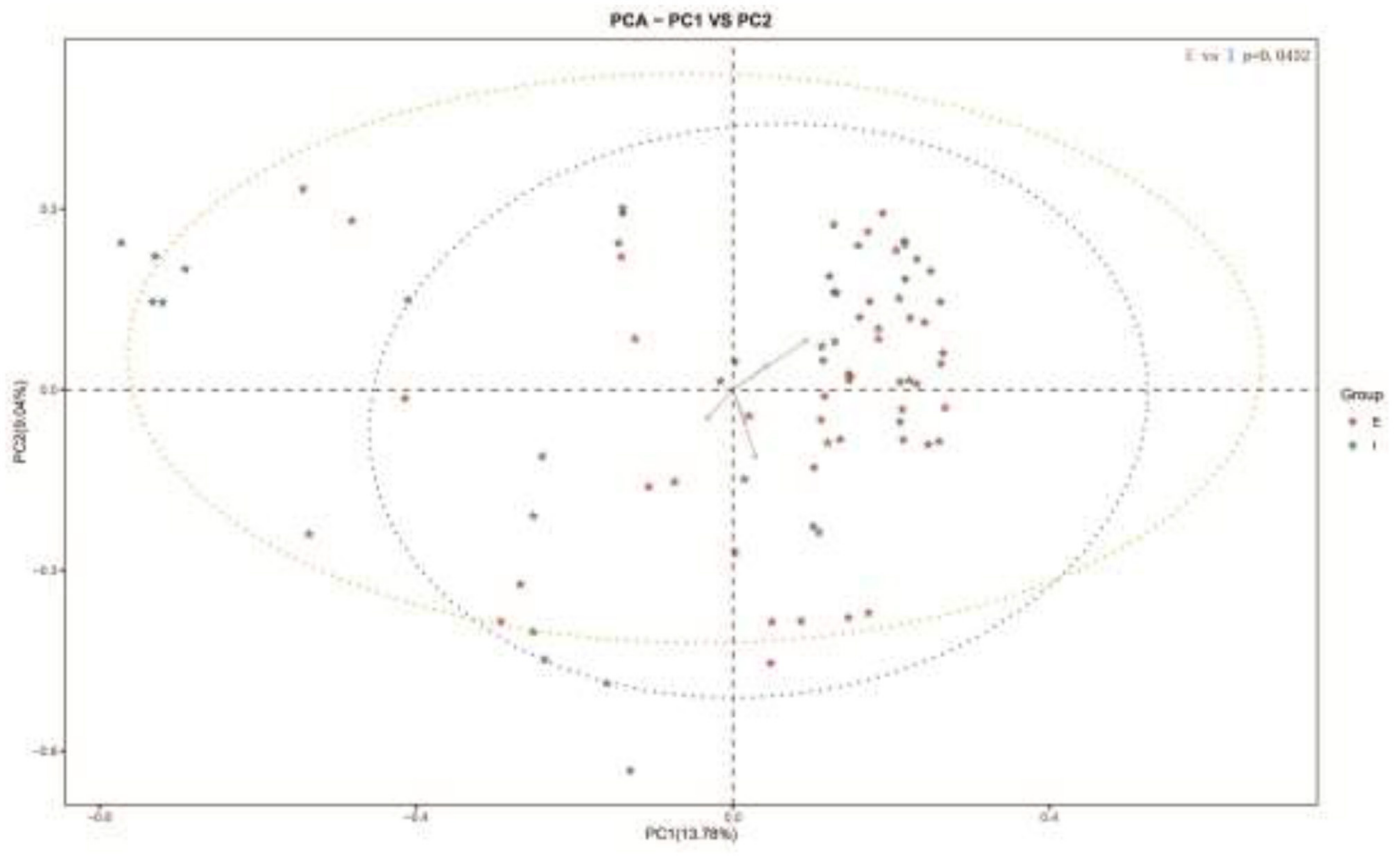
Beta analysis of microbial diversity in sputum samples of COPD frequent exacerbators and COPD infrequent exacerbators. E represented COPD frequent exacerbators; I represented COPD infrequent exacerbators. COPD, chronic obstructive pulmonary disease.

Subsequently, a Beta analysis of microbial diversity was performed. The results revealed that there were significant differences in the variety and quantity of microorganisms between COPD frequent and infrequent exacerbators (*p* < 0.05; Figure 2).

### Clustering of Bacterial Species

**Figure 3:**
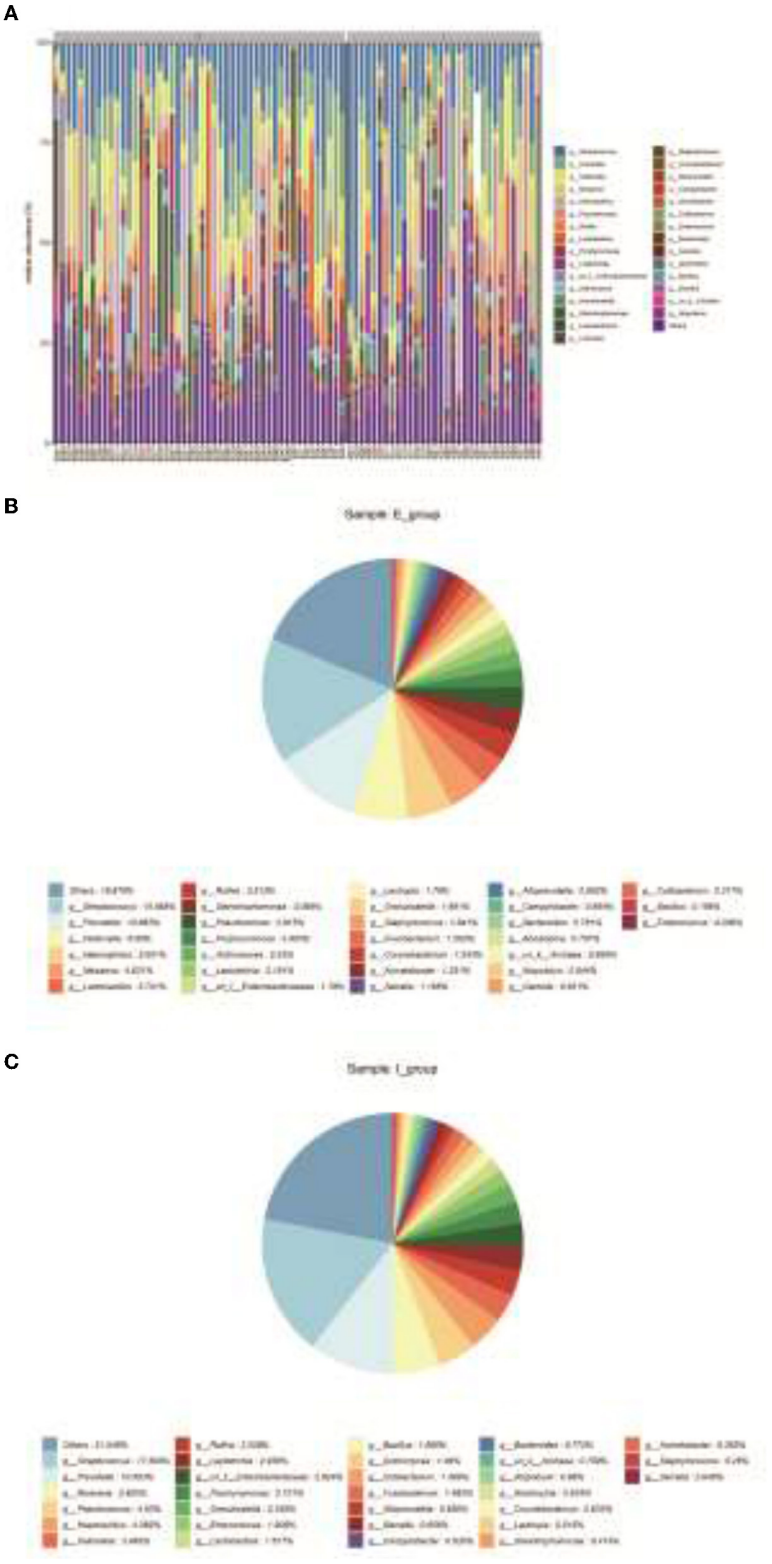
Clustering of bacterial species. **(A)**, Community compositions of both sample groups were analyzed and there were 30 primary bacterial species identified in the samples of the two groups. **(B)**, Relative abundance of intestinal dominant flora and cluster analysis of species in COPD frequent exacerbators at the genus level. **(C)**, Relative abundance of intestinal dominant flora and cluster analysis of species in COPD infrequent exacerbators at the genus level. E represented COPD frequent exacerbators; I represented COPD infrequent exacerbators. COPD, chronic obstructive pulmonary disease.

Community compositions of both sample groups were analyzed, and there were 30 primary bacterial species identified in the samples of the two groups (Figure 3A). Comparison of relative abundance of intestinal dominant flora and species clustering between the two groups at the genus level, it was found that the top five strains of COPD frequent exacerbators were g_Streptococcus (15.565%), g_Prevotella (10.683%), g_Veillonella (6.980%), g_Haemophilus (5.601%), and g_Neisseria (4.631%; Figure 3B). The first five strains of COPD infrequent exacerbators included g_Streptococcus (17.509%), g_Prevotella (10.6593%), g_Neisseria (5.925%), g_Pseudomonas (4.63%), and g_Haemophilus (4.382%; Figure 3C).

**Figure 4:**
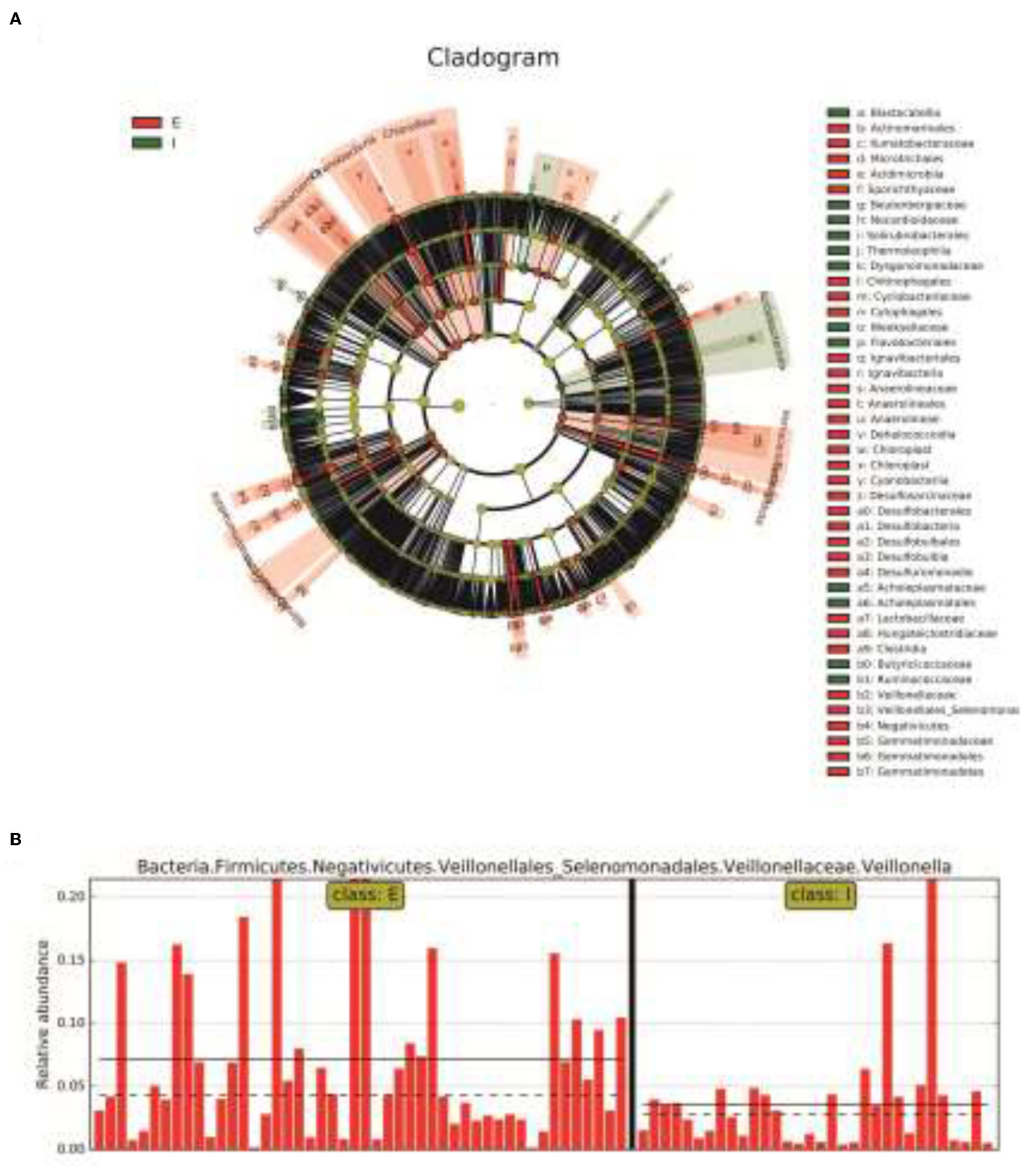
The LEfSe analysis was used to estimate the abundance influence of each strain on the difference. **(A)**, Cluster tree analysis. Red represents the group of COPD frequent exacerbators, green represents the group of COPD infrequent exacerbators, and nodes of different colors represent the importance of the microbiome in the representated group. The yellow nodes indicate the microbiota that did not play an important role in both groups. **(B)**, The abundance analysis of Veillonella in COPD frequent exacerbators and COPD infrequent exacerbators. E represented COPD frequent exacerbators; I represented COPD infrequent exacerbators. LEfSe, linear discriminant analysis effect size; COPD, chronic obstructive pulmonary disease.

Next, LDA Effect Size (LEfSe analysis) was performed to estimate the abundance influence of each strain on the effect of difference and to identify which colonies had a significant difference in sample classification. Figure 4A presents a clustering tree. Red represents the group of COPD frequent exacerbators, green represents the group of COPD infrequent exacerbators, and nodes of different colors represent the importance of the microbiome in the represented group. The yellow nodes indicate that the microbiota did not play an important role in both groups. Similarly, the relative abundance of Veillonella in COPD frequent exacerbators was higher than that of COPD infrequent exacerbators (Figure 4B).

### The Regulatory Effect of Veillonella on the Activity of Human Bronchial Epithelial Cells

**Figure 5:**
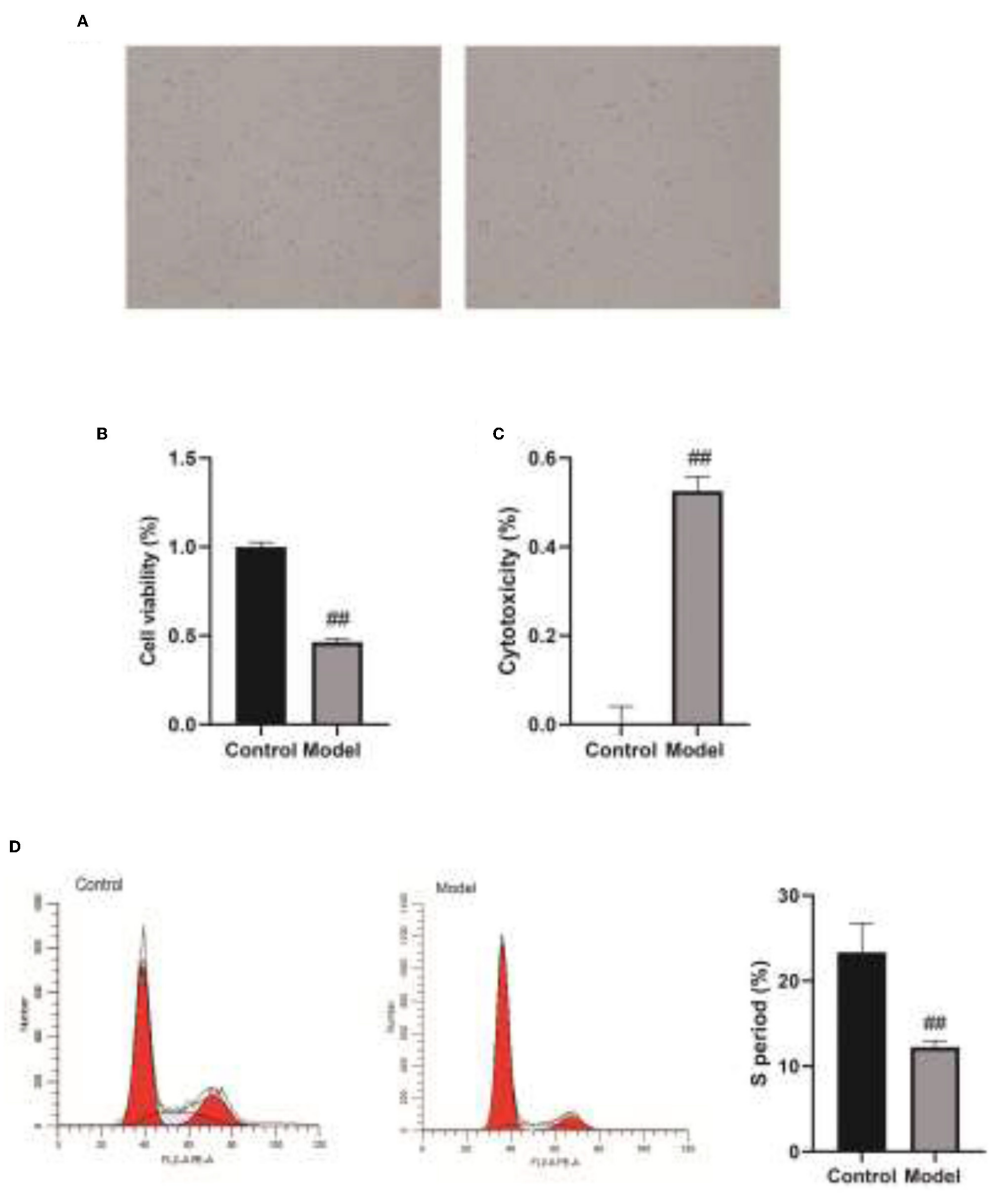
The regulatory effect of Veillonella on the activity of human bronchial epithelial cells. **(A)**, Human bronchial epithelial cells cultured *in vitro*. **(B)**, Cell viability detected by CCK8. **(C)**, LDH content determination. **(D)**, Cell cycles detection by flow cytometry. ##, *p* < 0.01. LDH, lactate dehydrogenase; CCK8, cell counting kit 8.

Subsequently, we added Veillonella parvula to human bronchial epithelial cells cultured *in vitro* (Figure 5A) and determined the regulatory effect of Veillonella parvula on human bronchial epithelial cells. The results indicated that Veillonella parvula markedly reduced the activity of human bronchial epithelial cells (*p* < 0.01; Figure 5B). The LDH content was increased substantially, indicating that Veillonella parvula produced apparent cytotoxicity (Figure 5C), causing cell membrane rupture and cell death. Furthermore, the results of flow cytometry indicated that the proportion of human bronchial epithelial cells in both the S phase and G2 phase decreased following Veillonella parvula treatment, indicating that Veillonella parvula inhibited cell proliferation (Figure 5D).

### Veillonella Parvula Regulates the Expressions of Inflammatory Factors

**Figure 6:**
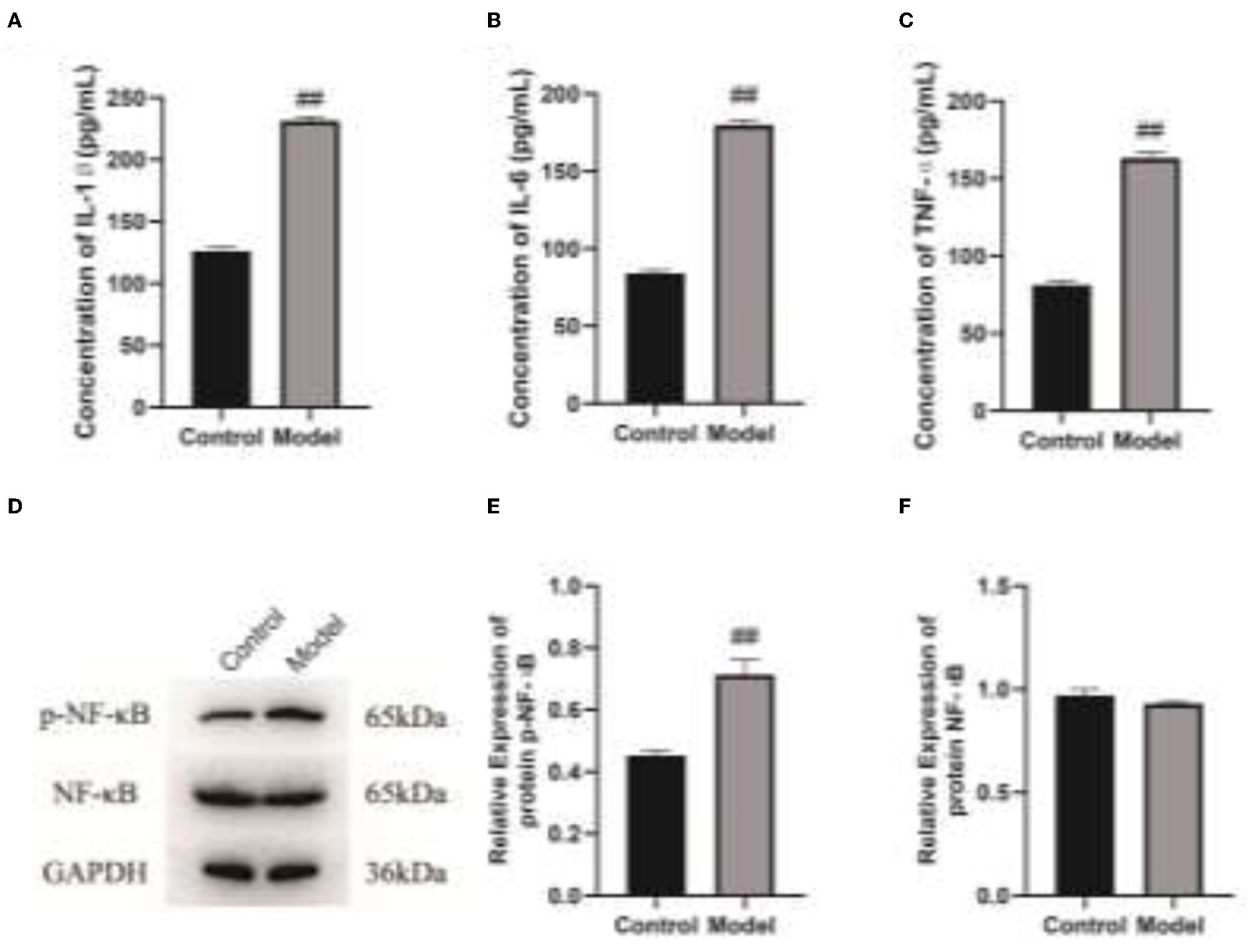
Veillonella parvula regulates the expressions of inflammatory factors. **(A–C)**, Expressions of IL-1, IL-6, and TNF-α detected by ELISA. **(D)**, The expressions of NF-κB and p-NF-κB detected by Western blot. **(E,F)**, Gray value analysis of NF-κB and p-NF-κB proteins. ## *p* < 0.01. IL-6, interleukin-L; TNF-α, Tumor Necrosis Factor α; ELISA, enzyme-linked immunosorbent assay.

To clarify the effect of Veillonella parvula on the expressions of cell inflammatory factors, ELISA and western blot assays were performed to detect the expressions of inflammatory factors. After Veillonella parvula treatment, the expressions of IL-1, IL-6, and TNF-α in cells were increased greatly (*p* < 0.01; Figures 6A–C), and the expression of p-NF-κB was increased significantly (Figures 6D–F), indicating that Veillonella parvula activated the inflammatory pathway and ultimately destroyed cell viability.

## Discussion

The mucosal epithelium of the respiratory tract is the first natural barrier of the body and the external environment, which is the first natural barrier against microbial infections. COPD is a frequent obstructive respiratory disease characterized by persistent respiratory symptoms and limited airflow due to airway obstruction (17, 18). As COPD has been reported as the third leading cause of death worldwide since 2020, the treatment of COPD acute exacerbators is of vital importance to the standardized management, prevention, and control of COPD (19). Bacterial or viral respiratory infections are the principal factor leading to acute exacerbation of COPD victims. This project aimed to study the distribution characteristics of flora in the respiratory tract among frequent and infrequent exacerbators in this region and to distinguish the inherent differences of flora in the respiratory tract between frequent and infrequent exacerbators. This study revealed that the Observed species index, Chao1 index, and ACE index of COPD frequent exacerbators were markedly higher than those of COPD infrequent exacerbators. The top five strains of COPD frequent exacerbators included g_Streptococcus (15.565%), g_Prevotella (10.683%), g_Veillonella (6.980%), g_Haemophilus (5.601%), and g_Neisseria (4.631%). We found that the relative abundance of Veillonella in COPD frequent exacerbators was higher than that of COPD infrequent exacerbators.

After the balance of the flora is broken, it will lead to changes in the local environment of the tissue. In addition, the increase or decrease of some flora can cause host infection and some other diseases. After the bacteria invaded tissue cells, they could replicate and survive in the cells. Meanwhile, some bacteria activated the cellular anti-apoptotic pathway, thereby evading immunity elimination of the host, which could directly induce the death of host cells and aggravate the damage of tissue cells. Veillonella represents an anaerobic gram-negative coccus, which can parasitize in the oral cavity, gastrointestinal tract, and female reproductive tract (20). Thirteen species of this genus have been identified, of which six species have been isolated from the human oral cavity. It has been reported that Veillonella is a copolymer with streptococcus (21). It was previously considered non-pathogenic and rarely caused serious infections. However, in recent years, there have been increasing reports of human infections in immunocompromised individuals. Statistics of the literature from 1976 to October 2015 have reported 31 cases of human infection with Veillonella. Of these cases, five cases were musculoskeletal infections caused by Veillonella parvula, such as four spinal infections. Additional reports also revealed cases of extramembranous abscess in patients with sclerocarcinoma affected by Veillonella parvula (22, 23). Veillonella parvula seems to be an opportunistic pathogen that affects immunocompromised patients. The present study indicated that the addition of Veillonella parvula to human bronchial epithelial cells cultured *in vitro* markedly reduced the viability of human bronchial epithelial cells. The LDH content in the cells was increased significantly, indicating that Veillonella parvula generated obvious cytotoxicity, which caused cell membrane rupture and cell death.

In addition, some studies have confirmed that COPD acute exacerbators are greatly affected by the persistent inflammation in the airway induced by bacterial infection (24, 25). Statistics results have indicated that the expression of IL-1β in COPD patients is increased, and the expression level of IL-1β is positively correlated with the increase of airway neutrophils and the decline of lung function index. The level of IL-1β in the airway (sputum) is recognized as a bacteria-associated biomarker of COPD acute exacerbators (26). Proteomic analysis of the sputum samples obtained from patients with COPD revealed that the proteomics characteristics of IL-1β-related sputum samples were identified. It also confirmed that the expressions of TNF-α and IL-6 were elevated and regulated by the IL-1β pathway. Elevated IL-1β and serum IL-6 expression levels in patients with COPD can activate the IL-1β-system inflammatory axis pathway and pose an increased risk for COPD frequent exacerbators (27, 28). The results of this study indicated that after Veillonella parvula treatment, the expressions of IL-1, IL-6, and TNF-α in the cells were elevated markedly, and the expression of p-NF-κB was increased substantially, indicating that Veillonella parvula activated the inflammatory pathway and ultimately destroyed cell viability. The findings also implied that Veillonella parvula could aggravate the response of airway inflammation, thereby promoting the susceptibility to COPD acute exacerbation.

Currently, the high-throughput sequencing of 16S amplicon is mainly introduced for the research on respiratory flora with a large sample size (29–31). This technique is only available for accurately identifying all bacterial components to the genus level. Luckily, the approach of metagenomic analysis can accurately determine the components of the microbial population, identify the functional areas of microorganisms combined with the macro transcriptome approach, and explore whether the entire microbial community can affect the metabolism of other microorganisms and/or hosts by generating signal molecules through the expression of specific genes, thereby further recognition of the signal transmission on the promotion of COPD occurrence and development. In the study of the microbiome, the concept of the brain-gut axis has been recognized by the public. COPD represents a systemic inflammatory disease of the lung affected by inflammation. The permeability of both the respiratory tract and intestinal mucosa may increase, which also provides the possibility for the migration or circulation of lung and intestinal flora. The previously described concerns will be potential research trends of respiratory flora in the future. This study has only verified the regulatory function of Veillonella parvula in cultured cells *in vitro* and has not been able to verify the function of more strains, nor has it been further verified in model animals. We know that the regulatory role of intestinal flora should be extensive, and only by understanding the regulatory network can we further formulate clinical medication and treatment plans. In future research, we will further verify the function of differentially expressed flora at the animal level and explore the possibility of improving the efficacy of clinically commonly used drugs or new drugs on COPD.

## Conclusion

The present study revealed that the relative abundance of Veillonella in COPD frequent exacerbators was higher than that of COPD infrequent exacerbators. Veillonella parvula activated the inflammatory pathway, ultimately destroyed the cell viability, and greatly impaired the activity of human bronchial epithelial cells, thereby inhibiting cell proliferation.

## Data Availability Statement

The data are deposited in https://submit.ncbi.nlm.nih.gov/subs/sra/, accession number SUB10905251.

## Ethics Statement

This research was a prospective and observational study, which was approved and documented by the Medical Ethics Committee of Chongqing the Seventh People's Hospital. The patients/participants provided their written informed consent to participate in this study.

## Author Contributions

LK, LC, and YY performed the experiment and collected the data. GC and LJ analyzed and interpreted the data. LK and ZC participated in writing this manuscript. All authors conceived and designed the study and approved this final manuscript.

## Funding

This work was funded by Chongqing Science and Technology Bureau of Banan District (No. 2020SHSY08).

## Conflict of Interest

The authors declare that the research was conducted in the absence of any commercial or financial relationships that could be construed as a potential conflict of interest.

## Publisher's Note

All claims expressed in this article are solely those of the authors and do not necessarily represent those of their affiliated organizations, or those of the publisher, the editors and the reviewers. Any product that may be evaluated in this article, or claim that may be made by its manufacturer, is not guaranteed or endorsed by the publisher.
