# Supplementary material for: Investigation on the Pathological Mechanism of Frequent Exacerbators With Chronic Obstructive Pulmonary Disease Based on the Characteristics of Respiratory Flora
Source: Front Med (Lausanne). 2022 Jan 20;8:816802. doi: 10.3389/fmed.2021.816802 (PMC8811034; doi:10.3389/fmed.2021.816802)
Supplement: Supplementary file 2 [file Data_Sheet_2.ZIP › 扫描全能王 2021-11-23 11.00(59).pdf]

知情同意书

尊敬的患者：

我们邀请您参加重庆市巴南区科委批准开展的《呼吸道微生物组研究应用于慢性阻塞性肺病频繁加重风险预测的模型构建》课题研究。本研究将在重庆市第七人民医院开展，估计将有约 90 名受试者自愿参加。本研究已经得到重庆市第七人民医院伦理委员会的审查和批准。本课题旨在初步研究重庆地区健康人群、FEs 和 IEs 患者气道菌群分布特征，区分 FEs 和 IEs 患者之间以及他们与健康人群间的气道菌群固有差异，我们设想气道定植的菌群是 COPD 病情加重的重要相关因素，以求找到与 FEs 相关的目标菌群作为 COPD 预后评估的生物标志物，为 COPD 频繁加重患者开辟一条新的防治途径。

研究中我该做什么

本研究随机选择 30 名无呼吸系统疾病的健康人（排除 1 个月内使用过抗菌药物者）以及符合 COPD 诊断标准的患者，其中患者包括 30 名 FEs（纳入标准：前 12 个月加重≥2 次）和 30 名 IEs，将 90 名研究对象分为 3 组，分别为：FEs 组、IEs 组和对照组。FEs 组和 IEs 组受试者于用药（包括抗菌药物和皮质类固醇类药物）前或停药后一个月，晨间清水漱口后取痰液于痰杯内待检。您只需配合研究医生提供相关病史资料，完成取样流程。研究还需要使用到一些您在住院期间的已经检查后剩余的血浆样本和相关的检查结果等信息。

参加该研究是否影响我的生活

该项研究于您主要涉及到无创取样，需要配合适当的时间留取痰液标本，其他方面不会额外影响您的生活。

我参加此研究会有什么风险和不良反应

该研究不涉及药物试验或有创取样，不会给您带来任何风险和不良反应。

从此研究中我能得到什么利益

本研究可以免费为您检测呼吸道菌群，对菌群进行生物信息学分

析，享有更加优化的临床治疗方案，研究结果可能对于 COPD 防治与预后评估有着深远的意义。

参加本研究会给予我什么报酬

您不会因参加本研究而获得任何酬劳。为了补偿您参加本研究可能给您带来的不便，我们会适量减免部分临床检验费用。

如果我在参加研究期间受到损害会怎样

一般情况下，该研究不会对您造成任何损害。即使您已经签署这份知情同意书，您仍然保留您所有的合法权利。

我的个人信息是保密的吗

您的医疗记录将保存在重庆市第七人民医院，研究者、研究主管部门、伦理委员会将被允许查阅您的医疗记录。任何有关本项研究结果的公开报告将不会披露您的个人身份。我们将在法律允许的范围内，尽一切努力保护您个人医疗资料的隐私。

关于您的个人和医疗信息将对外保密 且被保管在安全可靠的地方。在任何时候，您可以要求查阅您的个人信息，比如您的姓名和地址，如有需要可以修改这些信息。当您签署了这份知情同意书，代表您同意您的个人和医疗信息被用于上述所描述的场合

我必须参加研究吗

参加本研究是完全自愿的。您可以拒绝参加研究，或者研究过程中的任何时候选择退出研究，不需任何理由。该决定不会影响您未来的治疗。如果您不参加本研究，或中途退出不承担任何责任。

受试者同意声明

我已经阅读了上述有关本研究的介绍 对参加本研究可能产生的风险和受益充分了解。

我是自愿同意参加本文所介绍的临床研究。

我同意☑不同意□除本研究以外的其他研究利用我的医疗记录。

受试者签名：贺序秦　　　　　　　　联系电话：13399829770

法定代表人签名（如有）：　　　　　　联系电话：

与受试者关系：

日期：2020.6.11

研究者声明，我确认已向患者解释了本研究的详细情况　特别是参加本研究可能产生的风险和收益。

研究者签名：[illegible]　　　　　　　　联系电话：13508363817

日期：2020.6.11

重庆市第七人民医院
